# Exploring the taxonomical and functional profile of As Burgas hot spring focusing on thermostable β-galactosidases

**DOI:** 10.1038/s41598-020-80489-6

**Published:** 2021-01-08

**Authors:** María-Eugenia DeCastro, Michael P. Doane, Elizabeth Ann Dinsdale, Esther Rodríguez-Belmonte, María-Isabel González-Siso

**Affiliations:** 1 Grupo EXPRELA, Centro de Investigacións Científicas Avanzadas (CICA), Facultade de Ciencias, Universidade da Coruña, A Coruña, Spain; 2 Department of Biology, San Diego State University, 5500 Campanile Dr., San Diego, CA 92182 USA; 3 College of Science and Engineering, Flinders University, Sturt Rd, Bedford Park, SA 5042 Australia; 4 Syndey Institute of Marine Science, 19 Chowder Bay Rd, Mosman, NSW 2088 Australia

**Keywords:** Enzymes, Metagenomics

## Abstract

In the present study we investigate the microbial community inhabiting As Burgas geothermal spring, located in Ourense (Galicia, Spain). The approximately 23 Gbp of Illumina sequences generated for each replicate revealed a complex microbial community dominated by Bacteria in which Proteobacteria and Aquificae were the two prevalent phyla. An association between the two most prevalent genera, *Thermus* and *Hydrogenobacter*, was suggested by the relationship of their metabolism. The high relative abundance of sequences involved in the Calvin–Benson cycle and the reductive TCA cycle unveils the dominance of an autotrophic population. Important pathways from the nitrogen and sulfur cycle are potentially taking place in As Burgas hot spring. In the assembled reads, two complete ORFs matching GH2 beta-galactosidases were found. To assess their functional characterization, the two ORFs were cloned and overexpressed in *E. coli*. The pTsbg enzyme had activity towards o-Nitrophenyl-β-d-galactopyranoside (ONPG) and p-Nitrophenyl-β-d-fucopyranoside, with high thermal stability and showing maximal activity at 85 °C and pH 6, nevertheless the enzyme failed to hydrolyze lactose. The other enzyme, Tsbg, was unable to hydrolyze even ONPG or lactose. This finding highlights the challenge of finding novel active enzymes based only on their sequence.

## Introduction

Thermophiles, growing optimally at temperatures over 55 °C, are found in hot environments such as fumaroles, hydrothermal vents, hot springs, or deserts^1–4^. Apart from high temperatures, these habitats usually show other harsh conditions like extreme pH or high salt concentration. Therefore, the study of microorganisms inhabiting hot environments and their enzymes has drawn considerable interest from a biotechnological point of view, as these extremophiles have features suitable for industrial processes, in which high stability and activity at elevated temperatures, as well as high tolerance toward various reagents and solvents, are required.

The potential of thermal water as a source of novel thermostable biocatalysts has been demonstrated since a considerable number of thermozymes such as lipases^5^, polymerases^6^, or cellulases^7^, among others, have been isolated from hot springs. In recent years, metagenomics has become a powerful tool to explore the microbiological community composition and activity of extreme environments, like hot springs, whose conditions are difficult to reproduce in a lab-bench. The metagenomic approach is based on the study of the whole environmental microbial DNA (metagenome) that is directly sequenced, in what is called sequence metagenomics, or ligated into a vector and transformed to generate a metagenomic library, in what is known as functional metagenomics. Sequence metagenomics has enabled the study of a large number of hot springs extended all over the world like Tuwa, Lasundra and Unkeshwar hot springs in India^8–10^, a hot spring in Kamchatka, Russia^11^, Sungai Klah hot spring in Malaysia^12^ or several hot springs in Yellowstone National Park USA^13,14^.

β-galactosidases catalyze the hydrolysis of lactose to glucose and galactose, and they have drawn considerable interest from the biotechnological industry for the production of low-lactose milk and the revalorization of whey. Furthermore, some β-galactosidases can transfer the galactosyl residue of lactose carrying transgalactosylations reactions, which are frequently used for the synthesis of galacto-oligosaccharides (GOS), with prebiotic effects^15^, and to synthesize other galactosylated products^16^. Metagenomics has contributed to the exploration of heated habitats such as hot springs, either for ecological study or for bioprospection of novel enzymes. Some thermal β-galactosidases have been isolated from hot springs using functional metagenomics^17,18^ but there is only one reported study of thermostable β-galactosidases found in hot springs through sequence metagenomics^19^.

In the province of Ourense (Spain), there are at least 13 geothermal springs widespread across the region. Because of its accessibility and its historical importance, in this study, we have focused on As Burgas hot spring. Although some authors have previously investigated its water composition^20^, or its culturable microorganisms^21^, the present is the first reported metagenomic study of this hot spring. From the unassembled reads obtained through shotgun metagenomic DNA sequencing, we have assessed taxonomical and functional characteristics of As Burgas water population. Then, metagenomic sequences were assembled and annotated, finding two potential β-galactosidases that have been cloned, purified, and characterized.

## Results and discussion

### Taxonomic and functional assignment of metagenomic sequences

**Figure 1:**
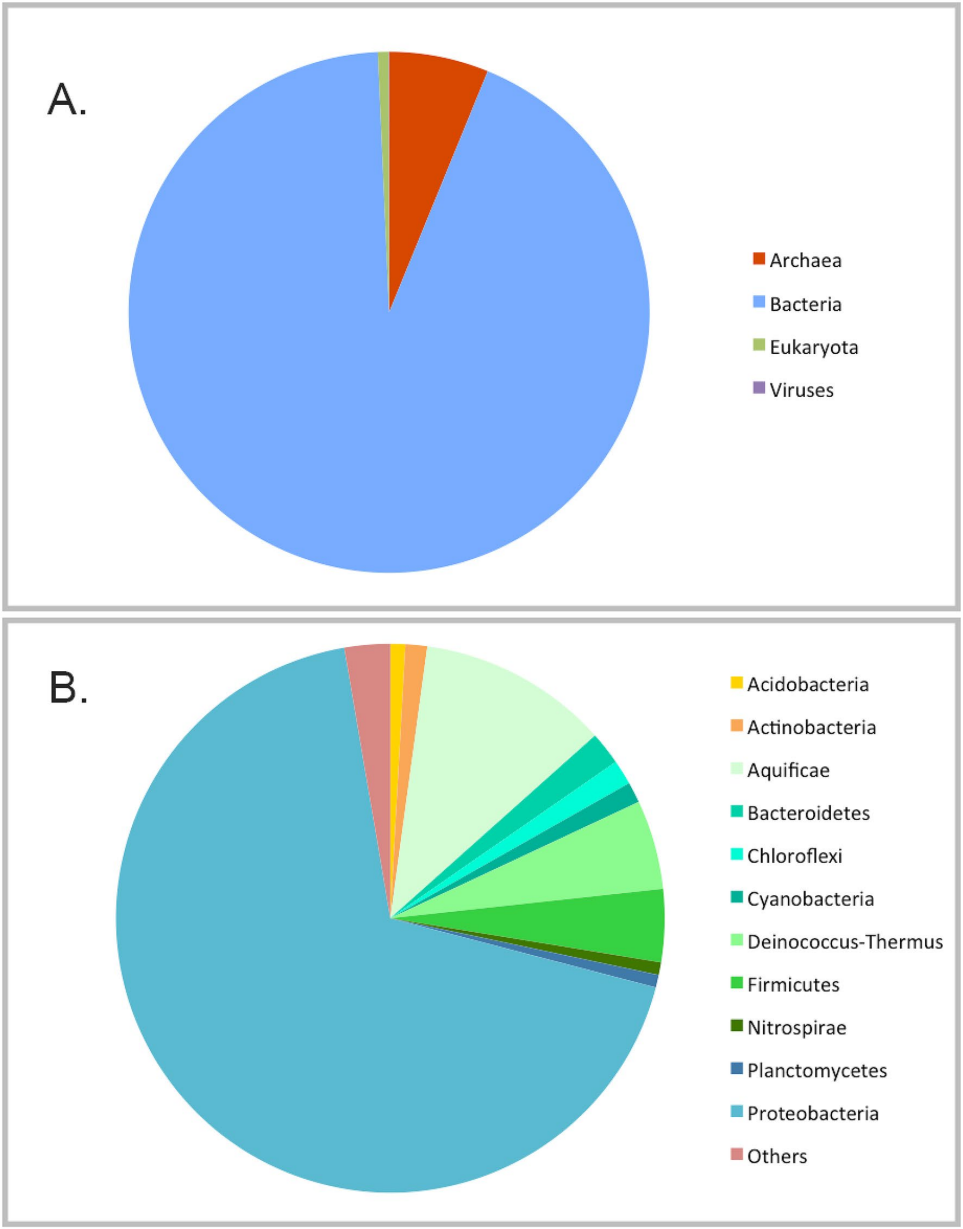
(**A**) Taxonomic assignment of the reads at domain level. The chart represents the percentage of reads assigned to each domain (relative abundance expressed as a percentage from the total assigned reads). (**B**) Taxonomic assignment of sequences within Bacteria domain. Percentage of reads annotated at phylum level is represented. Others include those phyla with less than 0.7% sequences assigned (Candidatus Poribacteria, Chlamydiae, Chlorobi, Chrysiogenetes, Deferribacteres, Dictyoglomi, Elusimicrobia, Fibrobacteres, Fusobacteria, Gemmatimonadetes, Lentisphaerae, Spirochaetes, Synergistetes, Tenericutes, Thermotogae, unclassified (derived from Bacteria) and Verrucomicrobia). Graphs were created with Excel for Windows version 14.0.0.

The BW1 and BW2 metagenomes consisted of 747,684 and 761,635 high quality reads, respectively (Table 1). There was no significant differences between the two samples (data not shown), thus the relative abundances of assigned reads to each taxon or function were expressed as an average. The taxonomical community analysis revealed a predominance of Bacteria (93.11 ± 1.86%), followed by Archaea (6.18 ± 1.84%), Eukaryota (0.67 ± 0.009%), and Viruses (0.02 ± 0.03%) (Fig. 1A). From the 27 bacterial phyla detected, the most abundant were Proteobacteria (68.25 ± 3.59%), Aquificae (11.24 ± 1.15%), Deinococcus-Thermus (5.26 ± 1.01%), Firmicutes (4.29 ± 0.53%) and Bacteroidetes (1.95 ± 0.19%) (Fig. 1B). More detailed information on the community structure is provided in supplementary material (Supplementary Tables S1, S2).

**Table 1:** Characteristics of the paired-end raw sequences obtained after Illumina MiSeq sequencing of As Burgas water before and after quality control (QC) with PRINSEQ.

	BW1		BW2		
	Read 1	Read 2	Read 1	Read 2	
**Before PRINSEQ QC**					
Number sequences	867,096	867,096	873,846	873,846	
Total bases	227,341,174	232,496,706	230,953,710	235,685,441	
Seq. length (bp)	262.19 ± 46.53	268.13 ± 47.31	264.30 ± 44.08	269.71 ± 45.04	
Mean GC content (%)	54.95 ± 11.23	55.32 ± 11.61	54.09 ± 11.76	54.46 ± 12.20	
Number of pairs	867 096 (100% sequences)		873 846 (100% sequences)		
**After PRINSEQ QC**					
Number sequences	747,684	747,684	761,635		761,635
Total bases	193,410,210	192,903,192	199,007,260		198,412,539
Seq. length (bp)	258.68 ± 46.62	258.00 ± 45.55	261.29 ± 43.84		260.51 ± 42.86
Mean GC content (%)	54.22 ± 11.41	54.51 ± 11.63	53.31 ± 11.87		53.60 ± 12.11
Number of pairs	747,684 (100% sequences)		761,635 (100.00% sequences)		

Read 1 and Read 2 correspond to the paired reads.

The predominance of Bacteria followed by Archaea was also found in the soil and the water of the Lobios hot spring, located in the same Galician region^22,23^. Nevertheless, in contrast with the significant relative abundance of Proteobacteria found in As Burgas water, Acidobacteria was the major phylum in the Lobios sediment while Deinococcus-Thermus dominated the Lobios water. These differences might be due to the influence of physicochemical parameters, such as pH and temperature, on the microbial community composition. In fact, As Burgas water has a lower temperature (66.3 °C) and pH (7.56)^20^ than Lobios water (76 °C, pH 8.2)^23^. It is also important to consider that taxonomical assignment in the study of Lobios water was done using assembled reads rather than the unassembled reads and thus, real phyla abundance might be lost^24^.

Temperature has been reported as a key factor in the prevalence of Proteobacteria. Dominance of this phylum has been found in geographically distant but moderate-temperature (29–65 °C) geothermal springs like Deulajhari and Tattapani in India^25,26^, Aguas Calientes in the Amazon rainforest of Perú^27^, Chiraleu, Ciocaia, and Mihai Bravu in Romania^28^ or El Coquito in the Colombian Andes^29^. Moreover, Power et al.^30^ found that the phyla Proteobacteria and Aquificae dominated in 925 geothermal springs in New Zealand (65.2% total average relative abundance across all springs), especially in hot springs with temperatures below 50 °C where Proteobacteria were the most abundant phylum. Similar results were found by Najar et al.^31^ that studied the microbial diversity of Polok (75–77 °C) and Borong (50–52 °C) hot springs in India finding that the dominance of the Phylum Proteobacteria was more pronounced in Borong hot spring, which had a lower temperature. Another distinctive aspect of Proteobacteria is that they are known to tolerate a higher concentration of sulfur and use reduced compounds of this element as an electron donor during their physiological processes^31^.

Aquificae is the second most abundant phylum in the As Burgas ecosystem consisting of 11.24 ± 1.15% of the metagenome. This phylum encompasses strictly thermophilic bacteria with an optimum growth temperature above 65 °C^32^. The high relative abundance of Aquificae occurs in other hot springs with a broad range of pH and temperatures, including six geothermal springs in the Philippines (60–92 °C, pH 3.72–6.58)^33^, the Mihai Bravu in Romania^28^ and the Ganzi Prefecture hot springs in China^34^. Members of this phylum dominate in environments with limited biomass and low ions concentrations, such as the King-Yu, Nono-Yu Koya, Yamanojo, and Jinata Onsen hot springs in Japan^35,36^, among others. Most Aquificae representatives are hydrogen-oxidizing bacteria that use hydrogen as electron donor, carbon dioxide as carbon source, and oxygen as the final electron acceptor. Alternatively, some species can oxidize thiosulfate or sulfur as energy sources^32^. Compared with other geothermal springs worldwide, the community structure of As Burgas is very similar to the Mihai-Bravu in Romania, which has similar temperature and pH (65 °C, pH 7.91)^28^, as both of the springs were dominated by phyla Proteobacteria, Aquificae, and Deinococcus-Thermus. This result suggests that chemolitotrophy by oxidation of H_2_ and reduced sulfur compounds are important metabolic processes in these springs and that the members of phylum Aquificae play a main role in primary productivity in this community.

Focusing on the genus level, the three most abundant genera in As Burgas water were *Thermus* (21,221 sequences (15.77%)), *Hydrogenobacter* (11,517 sequences (8.56%)) and *Thiobacillus* (5659 sequences (4.20%)). *Thermus* spp. has been traditionally described as heterotrophic thermophilic Gram-negative aerobic bacteria; although most are facultative anaerobes in the absence of oxygen and presence of nitrate^37^ but some species from the genera have shown the ability to grow mixotrophically^38,39^. The dominance of *Thermus* in As Burgas water is consistent with this genus’ optimal growth temperature (62–75 °C)^37^, in fact, members of this genus are commonly found in other thermal springs with temperatures above 60 °C. For example, in the hot springs of Heart Lake Geyser Basin in Yellowstone National Park, a shift in the microbial population was detected from several cyanobacterial genera at 44 °C to the observation of *Thermus* members at 63 °C and finally a predominance of this genus in the 75 °C geysers^40^. *Thermus* genus was also dominant in the 65 °C Mihai-Bravu spring in Romania^28^ and the Rupi Basin geothermal spring in Bulgaria^41^. This genus also dominates the water of the geographically close Lobios hot spring in Ourense^23^.

*Hydrogenobacter* was the second most abundant genus in As Burgas. These extremely thermophilic representatives of phylum Aquificae are obligate chemolithotrophic organisms with anaerobic anabolism, but aerobic catabolism^42^. High relative abundance and co-existence of *Hydrogenobacter* with *Thermus* genera was found in Lobios (Ourense)^23^, Rupi Basin (Bulgaria)^41^, Elegedi (Eritrea)^43^ and in Niujie (China)^44^ thermal springs. The association between hydrogen-oxidizing *Hydrogenobacter* with hydrogen-producing *Thermus* in these hot springs suggests hydrogen metabolism as an essential component of these ecosystems.

**Figure 2:**
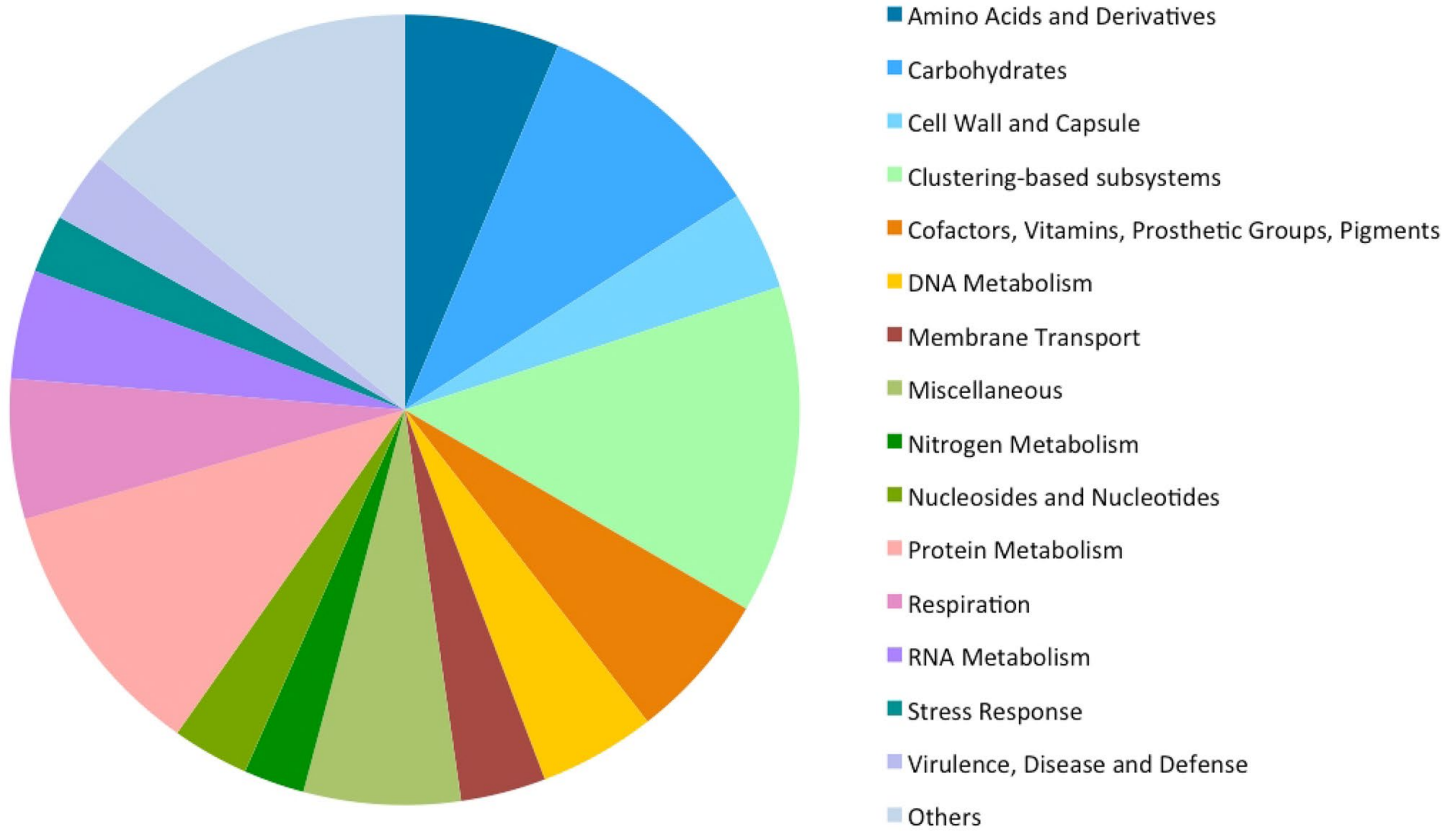
Functional profile of As Burgas hot spring at SEED subsystems level 1. Of 347,814 and 368,188 protein-coding regions predicted from BW1 and BW2 reads by MG-RAST, 52.1% (181,371) and 52.8% (194,410) were assigned by MG-RAST to SEED functional categories (Subsystems). The percentage of reads assigned to each function is represented. Others include those functions with less than 2.11% reads assigned (Cell Division and Cell Cycle; Dormancy and Sporulation; Fatty Acids, Lipids, and Isoprenoids; Iron acquisition and metabolism; Metabolism of Aromatic Compounds; Phages, Prophages, Transposable elements, Plasmids; Phosphorus Metabolism; Photosynthesis; Potassium metabolism; Regulation and Cell signaling; Secondary Metabolism; Sulfur Metabolism; Motility and Chemotaxis). Graph was created with Excel for Windows version 14.0.0.

In addition to the community analysis, functional analysis was performed with MG-RAST. The sequences that passed MG-RAST quality control produced 347,814 and 368,188 predicted protein-coding features for BW1 and BW2, respectively. From these, 52.1% (181,371) for BW1 and 52.8% (194,410) for sample BW2, were assigned annotation by MG-RAST to SEED functional categories (Subsystems) (Table 2). Among the functional categories at Level 1 identified by the SEED subsystems annotation, the four most dominant were the clustering-based subsystems (functional coupling evidence but unknown function; 13.44 ± 0.55%), protein metabolism (10.77 ± 0.17%), carbohydrates (9.55 ± 0.11%) and miscellaneous (6.42 ± 0.24%), based in the relative abundance of assigned reads (Fig. 2). More detailed information is provided in supplementary material (Supplementary Table S3). Similar results were found in Lobios hot spring water where the clustering-based subsystems were found as the largest category followed by miscellaneous, carbohydrates, and protein metabolism^23^. The predominance of the clustering-based subsystems in both metagenomes shows how limited our knowledge is regarding the functional annotation of the microbial proteome, as the precise functions of most proteins in metabolic pathways are yet to be revealed. Thus, the strategy of discovering new activities by a functional-driven metagenomic approach rises as a valid alternative to overcome such challenges.

**Table 2:** MG-RAST resume of the two replicates of As Burgas water metagenome (BW1 and BW2 samples MG-RAST Ids mgm4709017.3 and mgm4709018.3 respectively).

		BW1	BW2
Processed	Predicted protein features	347,814	368,188
	Predicted rRNA features	45,681	47,293
Alignment	Identified protein features	181,371	194,410
	Identified rRNA features	452	519
Annotation	Identified functional categories	152,744	163,890

Since O_2_ concentration is reduced in hot springs due to lower oxygen solubility in heated water, other electron acceptors are important, such as nitrate, elemental S, sulfate, or CO_2_. Thus, an overrepresentation of sequences related to nitrogen and sulfur metabolism could be expected in these kinds of habitats. Consequently, in this study, we specially review those pathways involved in nitrogen and sulfur metabolism.

Analysis of the nitrogen metabolism at subsystem level 3 revealed a high abundance of sequences involved in nitrate and nitrite ammonification, also known as dissimilatory nitrate reduction to ammonium (DNRA) (Table 3). DNRA is the result of anaerobic respiration by chemoorganoheterotrophic microorganisms using nitrate (NO_3_^−^) as a final electron acceptor, producing ammonia (NH_4_^+^). This metabolic pathway results in nitrogen (N) conservation in the ecosystems and is favored in habitats where NO_3_^−^ is limiting in relation to organic carbon^45^. Therefore, the low NO_3_^-^ content found in As Burgas water in comparison to other proximal geothermal springs such as Outariz, Tinteiro and Chavasqueira^20^ might be promoting the prevalence of DNRA bacteria like Proteobacteria^46,47^. This result is in accordance with the dominance of phylum Proteobacteria found in the taxonomical analysis of As Burgas metagenomic sequences. Nevertheless, it is important to remark that the presence or relative abundance of a gene in a metagenome does not mean that it is active. Metatranscriptomic studies are necessary to determine if DNRA is an important pathway in this ecosystem. In this aspect, other studies have reported the occurrence of an active DNRA pathway in some hot springs^48–50^.

**Table 3:** Analysis of Subsystems at level 3. From the 28 subsystems at level 3 registered by MG-RAST, only those subsystems with more than 2000 reads assigned were collected in the table.

SEED subsystems			No. of reads	
Level 1	Level 2	Level 3	BW1	BW2
Amino acids and derivatives	Branched-chain amino acids	branched-chain_amino_acid_biosynthesis	2799	2930
	Lysine, threonine, methionine, and cysteine	methionine_biosynthesis	3119	3134
Carbohydrates	CO_2_ fixation	Calvin-benson_cycle	2204	2351
	One-carbon Metabolism	Serine-glyoxylate_cycle	3311	3219
Cell Wall and capsule	NULL	Peptidoglycan_biosynthesis	2039	2106
Clustering-based subsystems	NULL	Bacterial_cell_division	2293	2119
Cofactors, vitamins, prosthetic groups, pigments	Tetrapyrroles	Heme_and_siroheme_biosynthesis	2244	2205
	Folate and pterines	YgfZ	2527	2741
DNA metabolism	DNA replication	DNA-replication	2446	2332
	DNA repair	DNA_repair,_UvrABC_system	2038	2019
	DNA repair	DNA_repair,_bacterial	2532	2661
Fatty acids, lipids, and isoprenoids	Fatty acids	Fatty_Acid_biosynthesis_FASII	2365	2459
Membrane transport	ABC transporters	ABC_transporter_branched-chain_amino_acid_(TC_3.A.1.4.1)	2598	2380
Motility and chemotaxis	Flagellar motility in prokaryota	Flagellum	3061	2874
Nitrogen metabolism	NULL	Ammonia_assimilation	2028	2279
	NULL	Nitrate_and_nitrite_ammonification	4965	4169
Nucleosides and nucleotides	Purines	De_novo_purine_biosynthesis	2868	3365
	Purines	Purine_conversions	2695	2672
Phosphorus metabolism	NULL	Phosphate_metabolism	3204	3396
Protein metabolism	Protein folding	Protein_chaperones	2584	2551
	Protein degradation	Proteolysis_in_bacteria,_ATP-dependent	2054	1933
	Protein biosynthesis	Ribosome_LSU_bacterial	4896	4258
	Protein biosynthesis	Ribosome_SSU_bacterial	3084	2958
	Protein biosynthesis	Universal_GTPases	2110	2012
Respiration	Electron donating reactions	Respiratory_complex_I	5524	5578
	Electron accepting reactions	Terminal_cytochrome_C_oxidases	3054	2826
RNA metabolism	Transcription	RNA_polymerase_bacterial	3475	3402
	RNA processing and modification	tRNA_modification_archaea	1801	2035
Sulfur metabolism	NULL	Sulfur_oxidation	3112	2665

A high number of reads with similarity to ammonia assimilation were found in As Burgas water metagenome (Table 3). The abundance of sequences annotated as glutamine synthetase and glutamate synthase, key enzymes in this metabolic pathway, were already expected as they are widely distributed among microorganisms, playing an important role in nitrogen metabolism^51^.

Reads annotated as Nitrogenase (*Nif*) genes for nitrogen fixation were also abundant in the metagenome. Although the distribution of these genes seems to be widespread in nature, as they have been described in different environments^52^ including hot springs^53–55^, active nitrogen fixation has been reported in several thermophilic organisms^56,57^. Nitrogen fixation could be important in As Burgas as this ecosystem harbors phyla with known diazotrophic representatives such as Proteobacteria and the phylum *Aquificae* in which some members of *Hydrogenobacter* were recently described as nitrogen-fixing bacteria^58^. Furthermore, nitrogen fixation has been demonstrated in other geothermal springs such as several hot springs from Yellowstone National Park^59,60^ and Nakabusa hot springs in Japan^61^, among others.

Nitrification might also take place in As Burgas ecosystem, as sequences matching the ammonia monooxigenase (AMO) enzyme were detected in the two metagenomes. This enzyme catalyzes the oxidation of ammonia to hydroxylamine and it is essential for chemolithotrophic ammonia-oxidizing bacteria. The oxidation of ammonia to nitrite in As Burgas hot spring water could be associated with the abundant Proteobacteria, as several members of this phylum have been described as autotrophic nitrifiers^62,63^.

Another important component in the nitrogen cycle is denitrification, which competes with DNRA, due to the dependence of both metabolic pathways on NO_3_^−^. Members of the genus *Thermus* are important denitrifiers in heated ecosystems, as they can perform facultative anaerobic respiration using NO_3_^−^ as the final electron acceptor, producing N_2_ or nitrous oxide (N_2_O)^37^. In addition, representatives from another abundant genus in As Burgas, *Thiobacillus*, also perform denitrification processes^64,65^. Unexpectedly, not many sequences related to denitrification were annotated in the metagenome (771 sequences in BW1 and 692 in BW2), even though these potential denitrifiers were two of the most abundant genera found in As Burgas. At the function level, sequences related to denitrification such as nitrite reductase (*nir*), nitric-oxide reductase (*nor*) and nitrous-oxide reductase (*nos*), were present in both metagenomes, but not in high abundance.

Functions involved in sulfur oxidation were also abundant in As Burgas water (Table 3). The high abundance of these sequences can be attributed to the prevalence of Proteobacteria in the microbial community, since this is an important sulfur-oxidizing phyla^66,67^. Numerous members of the abundant phylum Aquificae and Deinococcus-Thermus can oxidize thiosulphate or sulfur as an energy source and thus harbor *sox* genes^38,39,68^. Moreover, some sulfur-oxidizing bacterial species of the genus *Thermus* and *Thiobacillus* are also nitrate-reducing bacteria that accept electrons from the oxidation of reduced inorganic sulfur compounds and have been frequently identified in a diverse range geothermal springs^38,39,64^. Therefore, sulfur oxidation coupled with denitrification could be an important source of energy for carbon fixation in this hot spring, as was previously described for other hot springs^69^ and diverse heated habitats like hydrothermal vents^70^.

In relation to carbon-fixation metabolism, a high abundance of sequences associated with the reductive pentose phosphate cycle (Calvin–Benson cycle) (Table 3) was found. This cycle has been described as the principal pathway of carbon fixation in Cyanobacteria and Proteobacteria^71^ and some studies have reported the presence of genes related to this cycle in several *Thermus* strains^72^.

The number of sequences affiliated to the tricarboxylic acid (TCA) cycle was also representative (1742 for BW1 and 1775 for BW2), but slightly lower than those for the Calvin-Benson cycle. Most enzymes involved in the TCA cycle function in an oxidative way (releasing stored energy through the oxidation of acetyl-CoA into ATP and CO_2_), but they can be used by some microorganisms in a reductive TCA cycle that is essentially the oxidative TCA cycle running in reverse, leading to the fixation of two molecules of CO_2_ and the production of one molecule of acetyl-CoA^73^. Reverse TCA is suggested to be a more ancient pathway for carbon fixation^74^ and the main route for primary production at high temperatures (above 70 °C)^75^. The ability to perform the reverse TCA cycle is typical of bacteria from the phylum *Aquificae* such as *Hydrogenobacter*^75,76^ and was confirmed in a variety of anaerobic and microaerobic bacteria, including several proteobacteria^73^. Moreover, reads annotated as pyruvate: ferredoxin oxidoreductases (POR) were found in the two metagenomes. POR enzyme decarboxylates pyruvate to form acetyl-CoA and is crucial for the reverse TCA cycle, as it is able to act as pyruvate synthase catalyzing the reverse reaction^77,78^. The high abundance of sequences involved in the Calvin–Benson and reverse TCA cycles reveals that autotrophy is an important source of energy of the ecosystem, as was expected, in accordance with the low organic content of this kind of thermal habitats.

A high relative abundance of reads associated with one-carbon metabolism such as YgfZ, a folate-binding regulatory protein^79^ and sequences related to the serine-glyoxylate cycle (Table 3) were identified. Serine-glyoxylate cycle is a carbon assimilation pathway found in aerobic methanotrophs belonging to the classes Alpha-, Gammaproteobacteria, and the phylum Verrucomicrobia^80^. Sequences annotated as crucial enzymes for methanotrophic metabolism such as methane monooxygenase, methanol dehydrogenase or hydroxypyruvate reductase^81,82^ were present in the two replicates of As Burgas metagenome. A similar result was previously reported for the nearby Lobios hot spring, in which a high abundance of sequences associated with YgfZ and the serine-glyoxylate cycle was also detected. However, Lobios metagenome lacks the methane monooxygenase and methanol dehydrogenase encoding genes^23^. The methanogenic microorganisms frequently found in hot springs microbial mats^83^ would be the methane producers for methanotrophs in As Burgas. In fact, sequences annotated to the methanogenic orders Methanobacteriales, Methanocellales, Methanomicrobiales, Methanosarcinales, and Methanopyrales were found among the archaeal reads in the taxonomical analysis of As Burgas. Moreover, sequences matching several proteins involved in methanogenesis such as heterodisulfite reductase, formate dehydrogenase, and carbon monoxide dehydrogenase were found in the metagenome. Nevertheless, the presence of methyl-coenzyme M reductase gene, a key enzyme in methanogenesis^84^, was not detected in the metagenome.

### Sequence assembly and screening for sequences annotated as β-galactosidase

From the 873,846 quality paired-end BW2 raw reads, a total of 28,296 contigs with a maximum length of 263,962 bp and an average length of 932 bp (26,379,150 bp) were obtained using SPADes. From these, 26,417 sequences (93.36%) were annotated to the functional level with the MG-RAST. A search for β-galactosidase sequences with this tool resulted in only 2 sequences that harbor complete coding ORFs that were chosen for further study. Both selected ORFs belong to *Themus scotoductus* SA-01, as their nucleotidic sequence had 100% alignment with the *T. scotoductus* SA-01 complete genome, deposited in the GenBank by Gounder et al.^85^ under the accession number CP001962.1. This result is consistent with the dominance of *Thermus* genera reported in the taxonomical analysis. The deduced protein sequence of Tsbg and pTsbg consisted of 574 and 690 residues, respectively, and showed 100% homology with two different β-galactosidases from *T.scotoductus* with GeneBank accession number WP_015717803.1 and WP_015717801.1 for Tsbg and pTsbg respectively. The two proteins have been registered in GeneBank as part of a whole shotgun genome sequencing and annotation, but their cloning and expression have never been reported, therefore we selected both ORFs for further study and characterization. Both protein sequences contain a Glycosyl hydrolases family 2 (GH2) TIM barrel Domain (PF02836) according to Pfam protein database^86^. Therefore they are within the GH2 superfamily, in agreement with other thermostable microbial β-galactosidases like those from *Thermotoga maritima*^87^ or *Streptococcus thermophilus*^88^.

### Cloning, expression, and purification of *T. scotoductus* β-galactosidases

Both sequences were efficiently amplified, cloned in pDEST-527 vector and overexpressed in T7 Express *E. coli*. As no activity towards ONPG or lactose was detected for Tsbg, the gene was cloned in pDEST-527 without the histidine tag, in an attempt to discard the possibility of an incorrect folding or blocking of the active site due to the tag. Nevertheless, purified Tsbg protein without tag did not show activity using both lactose and ONPG as substrates. The lack of β-galactosidase activity in Tsbg is similar to the results obtained for *T. scotoductus* DSM 8553, as no β-galactosidase activity was detected in this strain^89,90^, which suggests that the cause is the protein itself rather than the expression host**.** Therefore, the successive characterization steps were only performed with the pTsbg.

### Effect of pH and temperature on activity and stability of recombinant pTsbg

**Figure 3:**
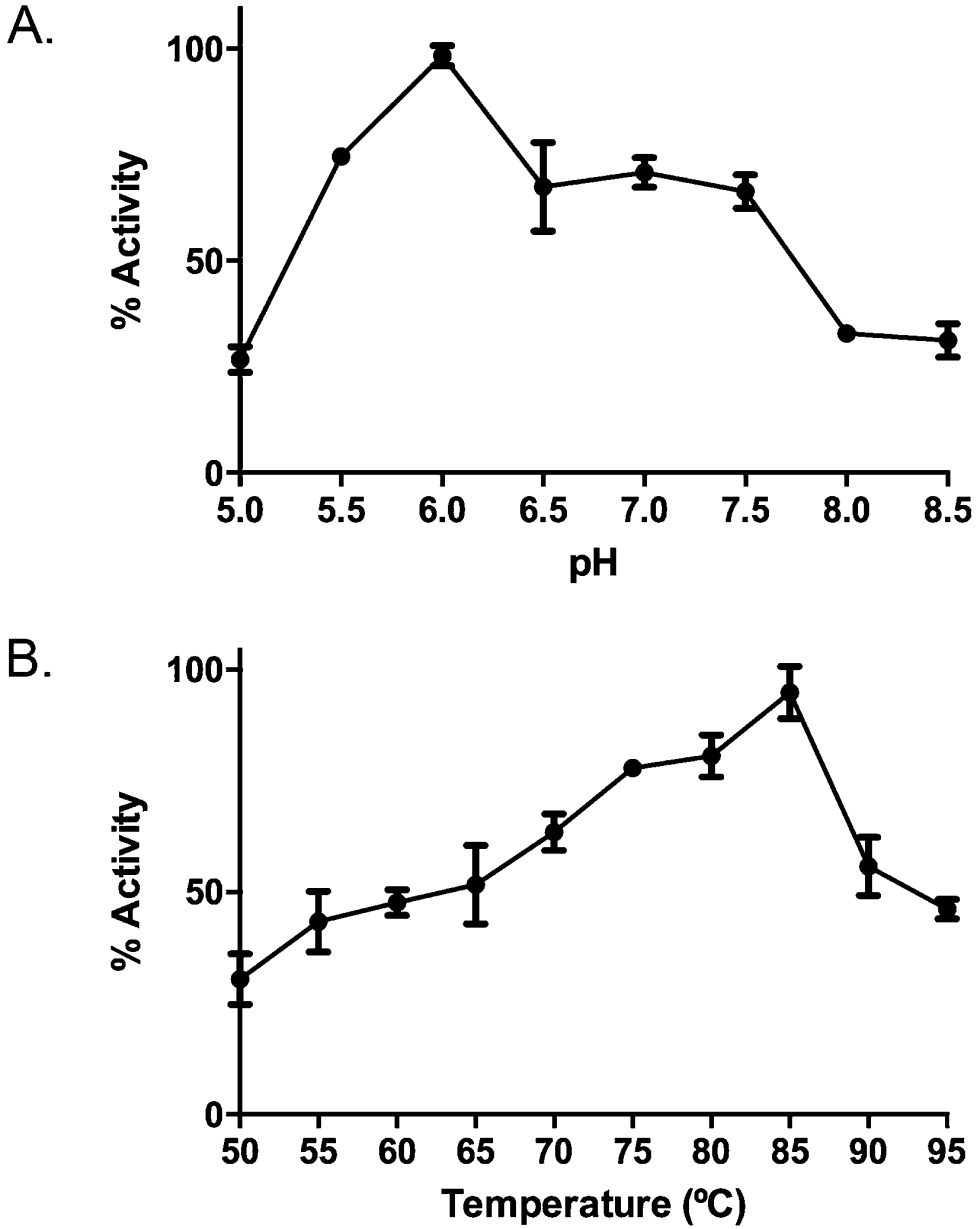
Effect of pH (**A**) and temperature (**B**) on the activity of pTsbg in Z Buffer using ONPG (4 mg mL^−1^) as substrate. Graphic was created using GraphPad Prism 6 for Windows (GraphPad Software, San Diego, California USA, www.graphpad.com).

pTsbg showed maximal activity at pH 6.0 in Britton-Robinson buffer using ONPG as substrate (Fig. 3A). This result is slightly lower than the optimum pH reported for other bacteria from *Thermus* genera like *T. thermophilus* HB8^91^, *T. thermophilus* HB27^92^ and it is comparable to the optimal pH reported for other thermostable β-galactosidases such as those from *Bacillus licheniformis*^93^, *Caldicellulosiruptor saccharolyticus*^94^, *Marinomonas* sp. BSi20414^95^ and much lower than the pH 7.8 reported for *T. oshimai* DSM 12092 β-galactosidase^96^.

As shown in Fig. 3B, maximal pTsbg β-galactosidase activity towards ONPG was found at 85 °C. This optimal temperature is higher than described using the same substrate for other counterparts of the genus *Thermus* such as *T.thermophilus* HB8^91^, *T.thermophilus* HB27^92^, *T. aquaticus* YT‐1^97^, *T. oshimai* DSM 12092^96^ and is the same reported as optimal to *T.thermophilus* KNOUC114 β-galactosidase^98^. When compared to other genera of thermophilic bacteria β-galactosidases, pTsbg showed higher optimal temperature than documented for the extremely thermophilic *C. saccharolyticus* and *Marinomonas* sp. BSi20414, which showed the optimum temperature at 80 °C and 60 °C respectively^94,95^. Nevertheless, the optimal temperature described for *Thermotoga naphthophila* RUK-10 β-galactosidase is higher^99^.

**Figure 4:**
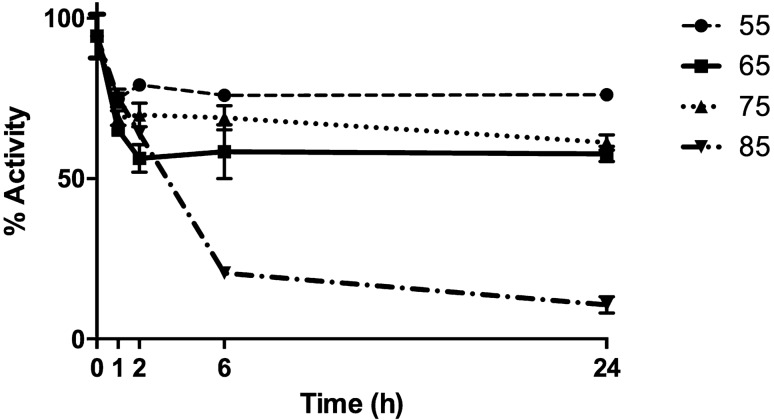
Effect of temperature on the stability of purified pTsbg. Graphic was created using GraphPad Prism 6 for Windows (GraphPad Software, San Diego, California USA, www.graphpad.com).

In relation to the thermal stability, pTsbg was able to retain up to 60% of its maximal activity towards ONPG after 24 h of incubation at 75 °C (Fig. 4).

### Determination of substrate specificity of pTsbg

Although substrate specificity of the enzyme was studied using the eight chromogenic substrates described in the “Methods” section, pTsbg was only active towards ONPG and p-Nitrophenyl-β-d-fucopyranoside. Moreover, the enzyme was unable to hydrolyze lactose and no transgalactosylation was observed in the presence of this substrate, as was determined by HPLC after carrying the reaction with 40% lactose at 70 °C and using a mix of galactose, glucose, lactose, raffinose and stachyose as standard (data not shown).

The preference for β-linked galactosidic substrates such as ONPG or p-Nitrophenyl-β-d-fucopyranoside over lactose has been frequently described in the characterization of β-galactosidases^99,100^. Similar to our results with pTsbg, other studies have reported β-galactosidases with activity towards ONPG but unable to hydrolyze their natural substrate lactose in vitro such as YesZ β-galactosidase from *Bacillus subtilis*^101^ or the β-Gal II from *Bifidobacterium adolescentis* DSM 20083^102^. The lack of β-galactosidase activity towards lactose reduces considerably the biotechnological potential of pTsbg, as it could not be applied to produce GOS from lactose and to generate lactose-free dairy products. Nevertheless, more studies focused on the fucosidase activity should be conducted, since pTsbg showed high activity with p-Nitrophenyl-β-d-fucopyranoside and may harbor fucosyltransferase activity that could be used for the synthesis of fucosylated oligosaccharides (FUCOS) with biological interest^103^ such as those from human milk.

## Conclusions

The taxonomical analysis of As Burgas hot spring metagenome reveals a microbial community dominated by Bacteria in which Proteobacteria (68.25 ± 3.59%) and Aquificae (11.24 ± 1.15%) are the most abundant phyla. The prevalence of the genera *Thermus* (15.77%) and *Hydrogenobacter* (8.56%) and the relation of their metabolism suggests an association between these two genera.

Moreover, the high relative abundance of sequences involved in the Calvin–Benson cycle and sequences annotated as key for the reductive TCA cycle unveils the dominance of an autotrophic population. Important pathways from the nitrogen and sulfur cycle such as DNRA, nitrification, or sulfur oxidation are potentially taking place in As Burgas hot spring, as was determined by the functional annotation of the metagenomic reads and in accordance with the microbial composition of the ecosystem.

After assembling the metagenomic reads two complete ORFs annotated as β-galactosidases were found. Both of them showed 100% homology with *T. scotoductus* SA-01 and were cloned and overexpressed in *E. coli*. The enzyme Tsbg lacked β-galactosidase activity using ONPG and lactose as substrates. On the contrary, pTsbg showed β-galactosidase activity towards ONPG but was not able to hydrolyze lactose; it showed β-fucosidase activity on the substrate p-Nitrophenyl-β-d-fucopyranoside, which suggests a priori unexpected biotechnological application. Once more this result reveals that the presence of a gene in a metagenome does not mean that it is active in the way predicted from the sequence, and highlights the importance of combining both functional and sequence metagenomics to find novel enzymes from metagenomes.

Our culture-independent study has provided an insight into the diversity of the microorganisms that inhabit As Burgas thermal environment, in an attempt to find novel β-galactosidases. Future research should be directed to characterize new environments, which will lead to better understanding of their ecological differences, and to find new enzymes of interest.

## Methods

### Sampling

Thermal water, with temperature 66.3 °C and pH 7.56^20^, was collected from As Burgas hot spring (GPS 42.334626, -7.865332), in Ourense (Galicia, Spain) in December 2015. Briefly, two samples (BW1 and BW2) of 50 L of water were collected into thermal water bottles, which were prewashed with 70% ethanol. The water sample was stored at room temperature until the next day when it was filtered through a nitrocellulose filter of 0.2 µm (25 L per filter and two filters per sample). Filters were preserved at − 20 °C until metagenomic DNA extraction.

### DNA extraction and shotgun sequencing

Total DNA was isolated from the filters using the Metagenomic DNA Isolation Kit for Water (Epicentre Biotechnologies; Madison, Wisconsin, United States), according to the manufacturer’s protocol. Metagenomic DNA of both replicates was quantified using Qubit dsDNA HS Assay kit (Invitrogen; Waltham, Massachusetts, USA) and prepared for Next Generation Sequencing using the Accel-NGS 2S Plus DNA Library Kit (Swift Biosciences; Ann Arbor, Michigan, USA). The amplified libraries were checked with a Bioanalyzer 2100 (Agilent Technologies; Santa Clara, California, USA) and concentrations were quantified by Qubit dsDNA HS Assay kit (Invitrogen). Paired-end sequencing of the metagenomic DNA libraries was performed with 2 × 300 bp using the MiSeq sequencer (Illumina, San Diego, California, USA) at San Diego State University.

### Taxonomic and functional assignment of metagenomic sequences

Illumina reads were treated with PRINSEQ software for quality control, removing all artificial duplicate reads and reads shorter than 60 base-pairs^104^. High-quality unassembled reads of both replicates were uploaded into the Metagenomics Rapid Annotation using the Subsystem Technology (MG-RAST) v4.0.3 server^105^ and are available under the accession numbers mgm4709017.3 (BW1) and mgm4709018.3 (BW2). MG-RAST is an automated annotation pipeline in which taxonomic assignment is done with BLAT comparisons^106^ to the NCBI, and gene functional potential with BLAT comparisons to the SEED protein database^105^. Sequence annotations were performed using the following parameters: cut off e-value 10^−5^, minimum 60% identity, and > 15 bp alignment length, as we have done previously^107^.

To reduce the differences related to library size, relative abundance was calculated as the percentage of reads assigned to a taxon or gene function in proportion to the total number of annotated reads.

### Sequence assembly and screening for sequences annotated as β-galactosidase

Paired-end unassembled high-quality reads were merged using PEAR^108^ and assembled with the SPAdes pipeline^109^. Then, assembled reads were uploaded to MG-RAST for functional annotation with the SEED subsystem database (maximum e-value of e−5, minimum identity of 60%, and minimum alignment length of 15). The contigs that contained β-galactosidases sequences were downloaded and analyzed for all possible open reading frames (ORFs) using NCBI ORF finder^110^. The ORFs and the deduced amino acid sequence were compared with other known sequences using nucleotide-nucleotide and protein–protein basic local alignment search tool (BLASTN and BLASTP) search^111^. The Pfam 32.0 web server, based on Pfam family database^86^ was used to infer the conserved domains within the amino acid sequences.

### Cloning, expression, and purification of *T. scotoductus* β-galactosidases

*Thermus scotoductus* β-galactosidase and putative β-galactosidase ORFs were amplified directly from the metagenomic DNA with the primers listed in Table 4 and both were cloned in the pDONR211 vector using the Invitrogen Gateway Technology (Invitrogen). From the Gateway vector, the gene was shuttled into the His-tagged expression vector pDEST-527, using the Gateway LR recombination reaction (Invitrogen). The constructions were transformed and expressed in T7 Express (C2566) *E. coli* (NEB). Induction was done with 0.4 mM IPTG for 2 h at 37 °C. Cells were collected by centrifugation (5000 rpm for 15 min 4 °C) and resuspended in 20 mM sodium phosphate buffer 500 mM NaCl (pH 7.2) and Complete Mini Protease Inhibitor Cocktail (Roche; Basel, Switzerland), following the manufacturer instructions. Cell disruption was done by sonication on ice using Vibra Cell sonicator (100 W, 5 min 2″ ON/8″ OFF) (Sonics & Materials; Newtown, Connecticut, USA). The resulting crude extract was preheated at 70 °C for 10 min to denature *E. coli* proteins, as suggested by Pessela et al.^112^. Then, the clear lysate obtained after centrifugation (14,000 rpm for 20 min) was passed through a HisTrap HP column (GEHealthcare; Chicago, Illinois, USA), following the manufacturers’ protocol and using an ÄKTA chromatography system (GEHealthcare). Briefly, the column was equilibrated with 20 mM sodium phosphate buffer 500 mM NaCl and 20 mM imidazole (pH 7.2) and the elution of the bound His-tagged fusion protein was done with a 20 mM sodium phosphate buffer 500 mM NaCl and 500 mM imidazole (pH 7.2). The selected fractions were concentrated and dialyzed using an Amicon Ultra-15 30,000 MWCO column (Millipore; Burlington, Massachusetts, USA). Purified protein concentration was quantified according to the Bio-Rad Protein Assay (Bio-Rad; Hercules, California, USA), employing bovine serum albumin as a standard. Protein samples of the different stages of the purification were run in a 10% SDS-PAGE gel for its molecular weight determination. NZYcolour Protein Marker II (Nzytech; Lisboa Portugal) was used as molecular weight standard and proteins were detected by staining with Coomassie Brilliant Blue.

**Table 4:** Primers used for the amplification of *T. scotoductus* β-galactosidase and putative β-galactosidase ORFs.

ORF amplified	Name	Sequence
*T.scotoductus* β-gal	MECA01f	5′ GGGGACAAGTTTGTACAAAAAAGCAGGCTTCATGAAGCTGGACCCCAACCATCCC 3′
	MECA02r	5′ GGGGACCACTTTGTACAAGAAAGCTGGGTCCTACTCCCAAAGCACCCGCCT 3′
*T.scotoductus* putative β-gal	MECA03f	5′ GGGGACAAGTTTGTACAAAAAAGCAGGCTTCATGAGGTGGGAAAGAGCTTGGTTTTTGG 3′
	MECA04r	5′ GGGGACCACTTTGTACAAGAAAGCTGGGTCTCACCAGGCCACCCCCAGG 3′

### Determination of β-galactosidase activity

Enzymatic activity was measured using ortho-Nitrophenyl-β-d-galactopyranoside (ONPG). Purified protein preparations were diluted in 150 µL Z buffer (100 mM Na_2_HPO_4_, 40 mM NaH_2_PO_4_, 10 mM KCl, 1.6 mM MgSO_4_, pH 7). Aſter incubation for 5 min at 85 °C, the reaction was started by adding 150 µL of a solution of 4 mg mL^−1^ ONPG in Z buffer to the enzyme preparation. Aliquots (100 µL) of the reaction mixture were stopped by adding 100 µL 1 M Na_2_CO_3_. Released o-nitrophenol was measured by UV absorbance at 420 nm. β-galactosidase activity is expressed in enzymatic units (U), defined as the amount of enzyme capable of releasing one µmol of the product (o-nitrophenol) per min (µmol min^−1^ mL^−1^) under the experimental conditions. All measurements were determined in triplicate.

### Effect of pH and temperature on activity and stability of recombinant pTsbg

To estimate the effect of pH on enzyme activity, the relative activities against ONPG (4 mg mL^−1^) were measured in the range of pH 5.0–8.5 using 20 mM Britton–Robinson buffer^113^. The influence of temperature was determined by measuring relative enzyme activities at 55–90 °C with ONPG (4 mg mL^−1^) in Z buffer. The thermal stability of the protein was assessed by pre-incubation of the enzyme in Z buffer at a range of 55–85 °C for different times followed by an activity assay against ONPG at 85 °C.

### Determination of substrate specificity and GOS production

The substrate specificity of the purified pTsbg was determined at 85 °C using 4 mg mL^−1^ solutions of the following chromogenic substrates in Z buffer (pH 7): ONPG, p-Nitrophenyl-β-d-fucopyranoside, p-Nitrophenyl-β-d-mannoside, p-Nitrophenyl-α-d-mannoside, p-Nitrophenyl-β-d-glucoside, p-Nitrophenyl-α-d-glucoside, p-Nitrophenyl-β-d-xyloside, and p-Nitrophenyl-α-d-xyloside.

GOS and lactose concentrations were determined by HPLC (HPLC Waters Breeze I), using a Waters Sugar-Pak column eluted at 90 °C with 0.1 M EDTA disodium salt in Milli-Q water at a flow rate of 0.5 mL min^−1^, and a Waters 2414 refractive-index detector. Purified protein was incubated at 70 °C and 650 rpm in phosphate buffer 0.1 M (pH 6.8), supplemented with 40% lactose. Samples were taken at 0, 0.5, 1, 2, 4, 6, and 24 h and immediately transferred to 99 °C for 5 min to inactivate the enzyme and stored at − 20 °C for subsequent analysis. Carbohydrates were quantified by external calibration, using standard solutions of galactose, glucose, lactose, raffinose, and stachyose.

## Supplementary Information

Supplementary Information.

## References

[1] Takai K (2004). *Thiomicrospira thermophila* sp. nov., a novel microaerobic, thermotolerant, sulfur-oxidizing chemolithomixotroph isolated from a deep-sea hydrothermal fumarole in the TOTO caldera, Mariana Arc, Western Pacific. Int. J. Syst. Evol. Microbiol..

[2] Nagata R (2017). *Lebetimonas natsushimae* sp. Nov., a novel strictly anaerobic, moderately thermophilic chemoautotroph isolated from a deep-sea hydrothermal vent polychaete nest in the Mid-Okinawa Trough. Syst. Appl. Microbiol..

[3] Amin A (2017). Diversity and distribution of thermophilic bacteria in hot springs of Pakistan. Microb. Ecol..

[4] Neveu J, Regeard C, Dubow MS (2011). Isolation and characterization of two serine proteases from metagenomic libraries of the Gobi and Death Valley deserts. Appl. Microbiol. Biotechnol..

[5] Meijide-Faílde, R., Leira, M., Torres Vaamonde, J.E., López Rodríguez, M. C. Estudio del componente biológico de las aguas mineromedicinales y termales de Ourense: Burgas y Outariz. In *Libro de actas del I Congreso Internacional del Agua "Termalismo y Calidad de Vida*, 519–524 (2016).

[6] Suharti, S., Hertadi, R., Warganegara, F. M., Nurbaiti, S. & Akhmaloka, A. Novel archaeal DNA polymerase B from Domas hot spring West Java. In *Proc. 5th International Seminar on New Paradigm and Innovation on Natural Sciences and Its Application (5th ISNPINSA), 2015*, Semarang, Indonesia. (2015).

[7] Zarafeta D (2016). Discovery and characterization of a thermostable and highly halotolerant GH5 cellulase from an Icelandic hot spring isolate. PLoS ONE.

[8] Mangrola A (2015). Deciphering the microbiota of Tuwa hot spring, India using shotgun metagenomic sequencing approach. Genomics Data.

[9] Mangrola AV, Dudhagara P, Koringa P, Joshi CG, Patel RK (2015). Shotgun metagenomic sequencing based microbial diversity assessment of Lasundra hot spring, India. Genomics Data.

[10] Mehetre GT, Paranjpe AS, Dastager SG, Dharne MS (2016). Complete metagenome sequencing based bacterial diversity and functional insights from basaltic hot spring of Unkeshwar, Maharashtra, India. Genomics Data.

[11] Eme L (2013). Metagenomics of Kamchatkan hot spring filaments reveal two new major (hyper)thermophilic lineages related to Thaumarchaeota. Res. Microbiol..

[12] Chan CS, Chan K-G, Tay Y-L, Chua Y-H, Goh KM (2015). Diversity of thermophiles in a Malaysian hot spring determined using 16S rRNA and shotgun metagenome sequencing. Front. Microbiol..

[13] Klatt CG (2013). Community structure and function of high-temperature chlorophototrophic microbial mats inhabiting diverse geothermal environments. Front. Microbiol..

[14] Colman DR (2016). Novel, deep-branching heterotrophic bacterial populations recovered from thermal spring metagenomes. Front. Microbiol..

[15] Panesar PS, Kaur R, Singh RS, Kennedy JF (2018). Biocatalytic strategies in the production of galacto-oligosaccharides and its global status. Int. J. Biol. Macromol..

[16] Wojciechowska A, Klewicki R, Sójka M, Grzelak-Błaszczyk K (2018). Application of transgalactosylation activity of β-galactosidase from *Kluyveromyces lactis* for the synthesis of ascorbic acid galactoside. Appl. Biochem. Biotechnol..

[17] Gupta R, Govil T, Capalash N, Sharma P (2012). Characterization of a glycoside hydrolase family 1 β-galactosidase from hot spring metagenome with transglycosylation activity. Appl. Biochem. Biotechnol..

[18] Schröder C, Elleuche S, Blank S, Antranikian G (2014). Characterization of a heat-active archaeal β-glucosidase from a hydrothermal spring metagenome. Enzyme Microb. Technol..

[19] Liu Z, Zhao C, Deng Y, Huang Y, Liu B (2015). Characterization of a thermostable recombinant β-galactosidase from a thermophilic anaerobic bacterial consortium YTY-70. Biotechnol. Biotechnol. Equip..

[20] González-Barreiro C, Cancho-Grande B, Araujo-Nespereira P, Cid-Fernández JA, Simal-Gándara J (2009). Occurrence of soluble organic compounds in thermal waters by ion trap mass detection. Chemosphere.

[21] Leira M, Meijide-Failde R, Torres E (2017). Diatom communities in thermo-mineral springs of Galicia (NW Spain). Diatom Res..

[22] Knapik K, Becerra M, González-Siso MI (2019). Microbial diversity analysis and screening for novel xylanase enzymes from the sediment of the Lobios Hot Spring in Spain. Sci. Rep..

[23] López-López O, Knapik K, Cerdán ME, González-Siso MI (2015). Metagenomics of an alkaline hot spring in Galicia (Spain): Microbial diversity analysis and screening for novel lipolytic enzymes. Front. Microbiol..

[24] Ju F, Zhang T (2015). Experimental design and bioinformatics analysis for the application of metagenomics in environmental sciences and biotechnology. Environ. Sci. Technol..

[25] Mohanrao Mahajan M (2016). Deciphering the microbial diversity of Tattapani hot water spring Using Metagenomic Approach. Int. J. Agric. Sci. Res..

[26] Singh A, Subudhi E (2016). Profiling of microbial community of Odisha hot spring based on metagenomic sequencing. Genomics Data.

[27] Paul S, Cortez Y, Vera N, Villena GK, Gutiérrez-Correa M (2016). Metagenomic analysis of microbial community of an Amazonian geothermal spring in Peru. Genomics Data.

[28] Chiriac CM (2017). Differences in temperature and water chemistry shape distinct diversity patterns in thermophilic microbial communities. Appl. Environ. Microbiol..

[29] Bohorquez LC (2012). In-depth characterization via complementing culture-independent approaches of the microbial community in an acidic hot spring of the Colombian Andes. Microb. Ecol..

[30] Power JF (2018). Microbial biogeography of 1,000 geothermal springs in New Zealand. BioRxiv..

[31] Najar IN, Sherpa MT, Das S, Das S, Thakur N (2018). Microbial ecology of two hot springs of Sikkim: Predominate population and geochemistry. Sci. Total Environ..

[32] Griffiths E, Gupta RS (2006). Molecular signatures in protein sequences that are characteristics of the phylum Aquificae. Int. J. Syst. Evol. Microbiol..

[33] Huang Q (2013). Archaeal and bacterial diversity in acidic to circumneutral hot springs in the Philippines. FEMS Microbiol. Ecol..

[34] Tang J (2018). Temperature-controlled thermophilic bacterial communities in hot springs of western Sichuan, China. BMC Microbiol..

[35] Nishiyama E (2018). The relationship between microbial community structures and environmental parameters revealed by metagenomic analysis of hot spring water in the Kirishima area, Japan. Front. Bioeng. Biotechnol..

[36] Ward LM (2019). Geochemical and metagenomic characterization of Jinata Onsen, a Proterozoic-analog hot spring, reveals novel microbial diversity including iron-tolerant phototrophs and thermophilic lithotrophs. BioRxiv..

[37] Cava F, Hidalgo A, Berenguer J (2009). *Thermus thermophilus* as biological model. Extremophiles.

[38] Skirnisdottir S, Hreggvidsson GO, Holst O, Kristiansson JK (2001). Isolation and characterization of a mixotrophic sulfur-oxidizing *Thermus scotoductus*. Extremophiles.

[39] Bjornsdottir SH (2009). *Thermus islandicus* sp. Nov., a mixotrophic sulfur-oxidizing bacterium isolated from the Torfajokull geothermal area. Int. J. Syst. Evol. Microbiol..

[40] De León KB, Gerlach R, Peyton BM, Fields MW (2013). Archaeal and bacterial communities in three alkaline hot springs in Heart Lake Geyser Basin Yellowstone National Park. Front. Microbiol..

[41] Tomova I (2010). Phylogenetic analysis of the bacterial community in a geothermal spring, Rupi Basin, Bulgaria. World J. Microbiol. Biotechnol..

[42] Pitulle C (1994). Phylogenetic position of the genus *Hydrogenobacter*. Int. J. Syst. Bacteriol..

[43] Ghilamicael AM, Budambula NLM, Anami SE, Mehari T, Boga HI (2017). Evaluation of prokaryotic diversity of five hot springs in Eritrea. BMC Microbiol..

[44] Bai S, Peng X (2019). Distinct microbial composition and functions in an underground high-temperature hot spring at different depths. Biogeosci. Discuss..

[45] Kraft B, Strous M, Tegetmeyer HE (2011). Microbial nitrate respiration—Genes, enzymes and environmental distribution. J. Biotechnol..

[46] Mohan SB, Schmid M, Jetten M, Cole J (2004). Detection and widespread distribution of the nrfA gene encoding nitrite reduction to ammonia, a short circuit in the biological nitrogen cycle that competes with denitrification. FEMS Microbiol. Ecol..

[47] Giacomucci L, Purdy KJ, Zanardini E, Polo A, Cappitelli F (2012). A new non-degenerate primer pair for the specific detection of the nitrite reductase gene nrfA in the genus *Desulfovibrio*. J. Mol. Microbiol. Biotechnol..

[48] Dodsworth JA, Hungate BA, Hedlund BP (2011). Ammonia oxidation, denitrification and dissimilatory nitrate reduction to ammonium in two US Great Basin hot springs with abundant ammonia-oxidizing archaea. Environ. Microbiol..

[49] Tripathy S, Padhi SK, Mohanty S, Samanta M, Maiti NK (2016). Analysis of the metatranscriptome of microbial communities of an alkaline hot sulfur spring revealed different gene encoding pathway enzymes associated with energy metabolism. Extremophiles.

[50] Alcamán-Arias ME (2018). Diurnal changes in active carbon and nitrogen pathways along the temperature gradient in porcelana hot spring microbial mat. Front. Microbiol..

[51] Nagatani H, Shimizu M, Valentine RC (1971). The mechanism of ammonia assimilation in nitrogen fixing bacteria. Arch. Mikrobiol..

[52] Dos Santos PC, Fang Z, Mason SW, Setubal JC, Dixon R (2012). Distribution of nitrogen fixation and nitrogenase-like sequences amongst microbial genomes. BMC Genomics.

[53] Klatt CG (2011). Community ecology of hot spring cyanobacterial mats: Predominant populations and their functional potential. ISME J..

[54] Jiménez DJ (2012). Structural and functional insights from the metagenome of an acidic hot spring microbial planktonic community in the Colombian Andes. PLoS ONE.

[55] Badhai J, Ghosh TS, Das SK (2015). Taxonomic and functional characteristics of microbial communities and their correlation with physicochemical properties of four geothermal springs in Odisha, India. Front. Microbiol..

[56] Wahlund TM, Madigan MT (1993). Nitrogen fixation by the thermophilic green sulfur bacterium *Chlorobium tepidum*. J. Bacteriol..

[57] Mehta MP, Baross JA (2006). Nitrogen fixation at 92 °C by a hydrothermal vent archaeon. Science.

[58] Nishihara A (2018). Nitrogenase activity in thermophilic chemolithoautotrophic bacteria in the phylum Aquificae isolated under nitrogen-fixing conditions from Nakabusa hot springs. Microbes Environ..

[59] Hamilton TL, Lange RK, Boyd ES, Peters JW (2011). Biological nitrogen fixation in acidic high-temperature geothermal springs in Yellowstone National Park, Wyoming. Environ. Microbiol..

[60] Loiacono ST (2012). Evidence for high-temperature in situ nifH transcription in an alkaline hot spring of Lower Geyser Basin, Yellowstone National Park. Environ. Microbiol..

[61] Nishihara A, Thiel V, Matsuura K, McGlynn SE, Haruta S (2018). Phylogenetic diversity of nitrogenase reductase genes and possible nitrogen-fixing bacteria in thermophilic chemosynthetic microbial communities in Nakabusa hot springs. Microbes Environ..

[62] Rotthauwe JH, Witzel KP, Liesack W (1997). The ammonia monooxygenase structural gene amoa as a functional marker: Molecular fine-scale analysis of natural ammonia-oxidizing populations. Appl. Environ. Microbiol..

[63] Stein, L. Y. & Nicol, G. W. Nitrification. In *eLS* 1–9 (Wiley, 2018). 10.1002/9780470015902.a0021154.pub2.

[64] Wood AP, Kelly DP (1988). Isolation and physiological characterisation of *Thiobacillus aquaesulis* sp nov, a novel facultatively autotrophic moderate thermophile. Arch. Microbiol..

[65] Yu L, Yuan Y, Chen S, Zhuang L, Zhou S (2015). Direct uptake of electrode electrons for autotrophic denitrification by *Thiobacillus denitrificans*. Electrochem. commun..

[66] Shao MF, Zhang T, Fang HHP (2010). Sulfur-driven autotrophic denitrification: Diversity, biochemistry, and engineering applications. Appl. Microb. Biotechnol..

[67] Watanabe T (2019). Genomes of neutrophilic sulfur-oxidizing chemolithoautotrophs representing 9 proteobacterial species from 8 genera. Front. Microbiol..

[68] Sano R (2010). Thiosulfate oxidation by a thermo-neutrophilic hydrogen-oxidizing bacterium, hydrogenobacter thermophilus. Biosci. Biotechnol. Biochem..

[69] Merkel AY (2017). Microbial diversity and autotrophic activity in Kamchatka hot springs. Extremophiles.

[70] Li Y (2018). Coupled carbon, sulfur, and nitrogen cycles mediated by microorganisms in the water column of a shallow-water hydrothermal ecosystem. Front. Microbiol..

[71] Kusian B, Bowien B (1997). Organization and regulation of cbb CO_2_ assimilation genes in autotrophic bacteria. FEMS Microbiol. Rev..

[72] Müller WJ (2016). Whole genome comparison of *Thermus* sp. NMX2.A1 reveals principal carbon metabolism differences with closest relation *Thermus scotoductus* SA-01. G3 Genes Genomes Genet..

[73] Hügler M, Wirsen CO, Fuchs G, Taylor CD, Sievert SM (2005). Evidence for autotrophic CO_2_ fixation via the reductive tricarboxylic acid cycle by members of the ε subdivision of proteobacteria. J. Bacteriol..

[74] Ragsdale SW (2018). Stealth reactions driving carbon fixation: New twists to bacterial metabolic pathways that contribute to the global carbon cycle. Science.

[75] Hügler M, Huber H, Molyneaux SJ, Vetriani C, Sievert SM (2007). Autotrophic CO_2_ fixation via the reductive tricarboxylic acid cycle in different lineages within the phylum Aquificae: Evidence for two ways of citrate cleavage. Environ. Microbiol..

[76] Ishii M (1998). Reductive TCA cycle in an aerobic bacterium, *Hydrogenobacter thermophilus* strain TK-6. Stud. Surf. Sci. Catal..

[77] Furdui C, Ragsdale SW (2000). The role of pyruvate ferredoxin oxidoreductase in pyruvate synthesis during autotrophic growth by the Wood-Ljungdahl pathway. J. Biol. Chem..

[78] Ikeda T (2010). Enzymatic and electron paramagnetic resonance studies of anabolic pyruvate synthesis by pyruvate: Ferredoxin oxidoreductase from *Hydrogenobacter thermophilus*. FEBS J..

[79] Teplyakov A (2004). Crystal structure of the YgfZ protein from *Escherichia coli* suggests a folate-dependent regulatory role in one-carbon metabolism. J. Bacteriol..

[80] But SY, Egorova SV, Khmelenina VN, Trotsenko YA (2019). Serine-glyoxylate aminotranferases from methanotrophs using different C1-assimilation pathways. Antonie van Leeuw. Int. J. Gen. Mol. Microbiol..

[81] Baik MH, Newcomb M, Friesner RA, Lippard SJ (2003). Mechanistic studies on the hydroxylation of methane by methane monooxygenase. Chem. Rev..

[82] Hanson RS, Hanson TE (1996). Methanotrophic bacteria. Microbiol. Mol. Biol. Rev..

[83] Schoenfeld TW (2013). Lateral gene transfer of family A DNA polymerases between thermophilic viruses, Aquificae, and Apicomplexa. Mol. Biol. Evol..

[84] Lyu Z, Shao N, Akinyemi T, Whitman WB (2018). Methanogenesis. Curr. Biol..

[85] Gounder K (2011). Sequence of the hyperplastic genome of the naturally competent *Thermus scotoductus* SA-01. BMC Genomics.

[86] El-Gebali S (2019). The Pfam protein families database in 2019. Nucleic Acids Res..

[87] Talens-Perales D, Polaina J, Marín-Navarro J (2016). Structural dissection of the active site of *Thermotoga maritima* β-galactosidase identifies key residues for transglycosylating activity. J. Agric. Food Chem..

[88] Geiger B (2016). From by-product to valuable components: Efficient enzymatic conversion of lactose in whey using β-galactosidase from *Streptococcus thermophilus*. Biochem. Eng. J..

[89] Yu T-T (2013). *Thermus tengchongensis* sp. nov., isolated from a geothermally heated soil sample in Tengchong, Yunnan, south-west China. Antonie Van Leeuw..

[90] Ullah Khan I (2017). *Thermus caldifontis* sp. nov. a thermophilic bacterium isolated from a hot spring. Int. J. Syt. Evol. Microbiol..

[91] MacIuńska J, Czyz B, Synowiecki J (1998). Isolation and some properties of β-galactosidase from the thermophilic bacterium *Thermus thermophilus*. Food Chem..

[92] Li, Y. *et al.* Study on the characterization of a potential thermostable β-galactosidase from *Thermus thermophilus* HB27. In *Proc. 2010 3rd International Conference on Biomedical Engineering and Informatics, BMEI 2010,* Vol. 5, 2118–2121 (2010).

[93] Jin HK, Yoon KH (2014). *Bacillus licheniformis* β-galactosidase. Korean J. Microbiol. Biotechnol..

[94] Park AR, Oh DK (2010). Effects of galactose and glucose on the hydrolysis reaction of a thermostable β-galactosidase from *Caldicellulosiruptor saccharolyticus*. Appl. Microbiol. Biotechnol..

[95] Ding H, Zeng Q, Zhou L, Yu Y, Chen B (2017). Biochemical and structural insights into a novel thermostable β-1,3-galactosidase from *Marinomonas* sp. BSi20414. Mar. Drugs.

[96] Gezgin Y, Tanyolac B, Eltem R (2013). Some characteristics and isolation of novel thermostable β-galactosidase from *Thermus oshimai* DSM 12092. Food Sci. Biotechnol..

[97] Berger J-L, Lee BH, Lacroix C (1997). Purification, properties and characterization of a high-molecular-mass b-galactosidase isoenzyme from *Thermus aquaticus* YT-I. Biotechnol. Appl. Biochem..

[98] Sook Nam E, Bong Choi H, Hyun Lim J, Jin Park H (2012). β-galactosidase-producing thermophilic bacterium, *Thermus thermophilus* KNOUC114: Identification of the bacterium, gene and properties of β-galactosidase. Int. J. Biol..

[99] Kong F, Wang Y, Cao S, Gao R, Xie G (2014). Cloning, purification and characterization of a thermostable β-galactosidase from *Thermotoga naphthophila* RUK-10. Process Biochem..

[100] Kang C-H (2011). A novel family VII esterase with industrial potential from compost metagenomic library. Microb. Cell Fact..

[101] Carneiro LABC, Yu L, Dupree P, Ward RJ (2018). Characterization of a β-galactosidase from *Bacillus subtilis* with transgalactosylation activity. Int. J. Biol. Macromol..

[102] Van Laere KMJ, Abee T, Schols HA, Beldman G, Voragen AGJ (2000). Characterization of a novel β-galactosidase from *Bifidobacterium adolescentis* DSM 20083 active towards transgalactooligosaccharides. Appl. Environ. Microbiol..

[103] Guzmán-Rodríguez F (2019). Employment of fucosidases for the synthesis of fucosylated oligosaccharides with biological potential. Biotechnol. Appl. Biochem..

[104] Schmieder R, Edwards R, Bateman A (2011). Quality control and preprocessing of metagenomic datasets. Bioinform. Appl. Note.

[105] Meyer F (2008). The metagenomics RAST server—A public resource for the automatic phylo-genetic and functional analysis of metagenomes. BMC Bioinform..

[106] Wilke A (2012). The M5nr: A novel non-redundant database containing protein sequences and annotations from multiple sources and associated tools. BMC Bioinform..

[107] Doane MP, Haggerty JM, Kacev D, Papudeshi B, Dinsdale EA (2017). The skin microbiome of the common thresher shark (*Alopias vulpinus*) has low taxonomic and gene function β-diversity. Environ. Microbiol. Rep..

[108] Zhang J, Kobert K, Flouri T, Stamatakis A (2014). PEAR: A fast and accurate Illumina paired-end reAd mergeR. Bioinformatics.

[109] Bankevich A (2012). SPAdes: A new genome assembly algorithm and its applications to single-cell sequencing. J. Comput. Biol..

[110] Wheeler DL (2003). Database resources of the National Center for Biotechnology. Nucleic Acids Res..

[111] Altschul SF, Gish W, Miller W, Myers EW, Lipman DJ (1990). Basic local alignment search tool. J. Mol. Biol..

[112] Pessela BCC (2004). A Simple strategy for the purification of large thermophilic proteins overexpressed in mesophilic microorganisms: Application to multimeric enzymes from *Thermus* sp. strain T2 expressed in *Escherichia coli*. Biotechnol. Prog..

[113] Britton HTS, Robinson RA (1931). CXCVIII. Universal buffer solutions and the dissociation constant of veronal. J. Chem. Soc..

